# Cyclicality of labour market search: a new big data approach

**DOI:** 10.1186/s12651-020-00283-9

**Published:** 2021-01-23

**Authors:** Christian Hutter

**Affiliations:** Institute for Employment Research (IAB), Nuremberg, Germany

**Keywords:** Big data, Labour market search intensity, Placement activity, Cyclicality, C55, J61, J64

## Abstract

This paper exploits big data on online activity from the job exchange of the German Federal Employment Agency and its internal placement-software to generate measures for search activity of employers and job seekers and—as a novel feature—for placement activity of employment agencies. In addition, the average search perimeter in the job seekers’ search profiles can be measured. The data are used to estimate the behaviour of the search and placement activities during the business and labour market cycle and their seasonal patterns. The results show that the search activities of firms and employment agencies are procyclical. By contrast, job seekers’ search intensity shows a countercyclical pattern, at least before the COVID-19 crisis.

## Introduction

Standard search and matching theory (e.g. [21]) states that labour market matches are formed using unemployed and vacancies, and an efficiency parameter describing how well unemployed and open positions form matches. In this context, the efficiency parameter covers a range of factors such as mismatch [11, 22] or information and institutions. A key ingredient of matching efficiency, however, is the behaviour of the relevant agents: Whether people get into work, for example, depends crucially on how intensively unemployed look for jobs and how much effort employers make when trying to fill an open position. This behaviour can be described as search intensity. A further crucial factor of matching efficiency is flexibility and the willingness to make some compromise during the search for a job or a suitable candidate.

Beyond job seekers and employers, a further agent is present in the labour market: the employment service (see e.g. [10]). The activities of the intermediary between the two sides can have an impact on how quickly unemployed and vacancies come together. In many countries, the public employment service is the central point of contact for unemployed and firms as it provides support to form matches between both market sides. However, while there are some studies on search intensity of unemployed [5, 20] and firms [2, 3], there are no empirical measures of placement intensity of employment agencies so far, despite its importance. In Germany, for instance, the relevance of this intermediary showed itself during the COVID-19 crisis, when it was impossible for employment agencies and job centers to pursue their usual placement activities under corona conditions due to other priorities such as massively increasing short-time work.

This paper contributes to the literature by measuring search intensity using a source of big data that directly captures online activity: It evaluates how often the job exchange website of the German Federal Employment Agency (FEA) and its placement platform have been accessed by job seekers and firms for search activities. In this context, “big data” refers to the millions of visits per month on the FEA’s online job exchange that are processed and transformed into aggregate search measures in this paper. Furthermore, it takes a closer look on the average search perimeter from the job seekers’ search profiles which they can submit on the online job exchange platform. The idea is that a higher search radius could signal a higher willingness to make some compromise so that it can serve as a potential proxy for the job seeker’s flexibility.

As a counterpart to search intensity, the notion of placement intensity of the labour market intermediary is introduced. The data at hand allow—for the first time—measuring placement activity of employment agencies. For this purpose, online activity at *VerBIS*, the FEA’s internal placement software, is exploited. With this software, employment agents perform genuine placement activities. For instance, they screen the labour market to find suitable candidates for job openings (or vice versa) and generate a placement proposal. To my knowledge, the placement activities of employment services have not yet been investigated. Consequently, having a measure of the central matching process on the labour market is all the more valuable.

A central contribution is to empirically analyse important time variation properties of the novel search activity measures. This extends previous literature: While [1], for instance, find matching efficiency as a whole to be procyclical, this paper aims to investigate the cyclical behaviour of several key factors of matching efficiency: firms’ and job seekers search intensity, placement intensity, as well as job seekers’ search perimeter. Several studies analyse job seekers’ search intensity (e.g. [5, 20]). Moreover, [3] consider recruiting intensity of firms and find procyclical recruitment intensity. Employers increase their hiring efforts in stronger, and thus tighter, labour markets in order to fill their positions. In contrast, search intensity of the unemployed is often found to be countercyclical. While lower chances to receive a job offer in times of labour market slackness should discourage search, the fact that search must be intensified in downturns to compensate for lower job arrival rates works in the opposite direction [19]. Furthermore, if average characteristics worsen during economic booms, countercyclicality could be reinforced [20].

While there is at least some ambiguity in the literature on the cyclical behaviour of search intensity, the intermediary’s behaviour in the course of a business or labour market cycle has not been investigated so far. One could think of several mechanisms at work here. If a tighter labour market requires employment agents to more intensively support firms in their search for suitable candidates, placement intensity could behave procyclically. By contrast, the agency might intensify support for unemployed and placement activities in times of economic downturn. Characteristics of the unemployed could play a role, too. If, for example, there are more unemployed with low job opportunities, the agency might intensify support, especially for difficult cases. However, this could then come at the expense of support for “easier” cases, given a limited working time of the employment agents. In the end, the cyclical behaviour of the search and placement activities is an empirical question that will be investigated in this paper.

In a comprehensive explorative investigation using correlation and regression analysis, the study contrasts search and placement activities with GDP and labour market tightness. The results show that firms and employment agencies display pro-cyclical search patterns. By contrast, job seekers’ search intensity and search perimeter increases during times of weaker economy and labour market. In general, the cyclical behaviour is confirmed also when a broad set of control variables is included in the regressions.

Beyond the cyclical movements, the paper also analyses the seasonal patterns of the search and placement activities. They might provide explanations for seasonal patterns in aggregate unemployment or employment. The results show that the seasonal patterns closely align with the main holiday seasons in winter and (to a less pronounced extent) in summer, and with the vocational training cycle.

The paper is structured as follows: The next section focuses on how the novel data are obtained. Section 3 discusses theoretical considerations. Section 4 presents the results on cyclicality and seasonality. Section 5 focuses on search and placement during the COVID-19 pandemic. The final section concludes.

## Measuring search and placement activities from big data

Conventional measures of search activity are often based on survey data, e.g. from time-use surveys [8, 14, 20]. With the digitalisation of labour markets, online data such as the presence of individuals in online job search [7, 15] or applications to job postings [4] got into the focus. However, the job exchange of the FEA and its placement-software *VerBIS* open up innovative big data sources for research. This section focuses on how these sources are exploited for measuring search and placement activities.

The FEA as the central intermediary for the unemployed runs a job exchange website^1^ where job seekers (*JS*) can apply for open positions or offer their workforce, and firms (*F*) can find workers or place job offers. Once the job exchange is accessed, server log files are stored in anonymous form.^2^ These server log files are then processed in Netmind, a software that allows accessing the data without publication lag. The data in Netmind provide valuable information, e.g. about which part of the job exchange website the user has visited. Thus, they allow distinguishing whether the job seekers’ or employers’ area of the job exchange was accessed and hence measuring the respective search intensities ($I^{JS}$, $I^{F}$). For instance, if the specific URL visited implies that the visitor wanted to look over her job openings or to find suitable job candidates, the exchange website was most likely accessed by an employer. On the other hand, if, for instance, the visitor searched for suitable job openings, it can be assumed that a job seeker accessed the website.

Throughout the paper, “activated visits” are used, i.e. only online activities where a visitor was active on the website beyond merely opening it are counted.^3^ Since activated visits involve more than one page view, it can be assumed that the visitor is interested in the content and took a closer look at it. Thus, activated visits represent the qualified traffic on the online job exchange platform. Furthermore, this helps exclude unwanted online traffic, e.g. by bots, from the data.

In addition to search data of the FEA’s job exchange, Netmind also provides access to data of *VerBIS*, the FEA’s internal placement software. With this software, employment agents (*EA*) screen the labour supply and demand sides to identify potential positions for job seekers or suggest candidates for an open position. It is important to know that VerBIS and the FEA’s online job exchange are two different platforms, i.e. the placement procedure is performed by the employment agents using VerBIS irrespective of how active the job seekers or firms are at the online job exchange. The placement procedure can be broken down into single tasks starting with the first contact with the unemployed person and ending with postprocessing after the employment agent has issued a placement proposal. All of these single steps must be documented in VerBIS and hence are comprised in the aggregate online data at hand. Since administrative tasks are also carried out in VerBIS, the information in Netmind serves to identify genuine placement activities (e.g. generating a placement proposal) to measure the placement intensity $I^{EA}$. To my knowledge, the placement activities of employment services have not yet been investigated.

To generate monthly data of online activity, the counting days of the FEA’s statistics were used. Hence, the way the search activities are measured is consistent with headline figures of the FEA such as unemployment or vacancies. A counting day typically is around the middle of a month.^4^ In order to capture search and placement *intensities* instead of mere accumulated *activities*, the sum of activated visits between two counting days is divided by either the number of unemployed (in case of $I^{JS}$), the number of vacancies (in case of $I^{F}$), or by the sum^5^ of unemployed and vacancies (in case of $I^{EA}$). Note that throughout this paper, “search intensity” is used synonymous to activated visits of job seekers *per unemployed*, or activated visits of firms *per vacancy*. The question whether this search intensity can be explained by compositional effects rather than actual changes in search *behaviour* is treated in Sect. 4.3.

The three intensity measures are calendar-adjusted, i.e. divided by the number of working days between two counting days, and seasonally adjusted. While Sects. 4.1 to 4.4 work with seasonally adjusted data only, Sect. 4.5 treats the seasonal patterns of the novel data in more detail. Occasionally, there are missing data due to changes in the platforms. Potential structural breaks after periods of missing data are eliminated by level shift dummies in ARMA models.

Throughout this paper, the search and placement measures are calculated counting all relevant activities. For instance, all activated visits at the job seekers’ part of the job exchange are added up. An alternative would be to look at the most important single activities performed at the job exchange. So instead of counting all online activities of the job seekers at the job exchange, for instance, one could count only how often the specific URL “search for job offers” or “display job offers” was accessed. It is possible that such alternative measures are subject to different time series dynamics. However, this approach sounds more promising than it eventually turns out to be: The data show virtually the same dynamics so no major changes can be expected: The correlation of “search for job offers” and “display job offers” with the job seekers’ total online activities is r = 0.99 in both cases. This phenomenon is similar when looking at the VerBIS data. Here, instead of summing up all placement activities of the employment agents, one could look again only at single URLs (for instance the one connected to the task “generating a placement proposal”). However, again the correlation is never less than r = 0.9, which is no surprise since most of the necessary steps in the placement process are highly standardized, i.e. regularly performed, and must be documented in VerBIS.

On the FEA’s job exchange, a job seeker can also create a profile for job searching purposes. Besides information about the desired job and the applicant’s educational attainments and skills, there is the possibility to enter a search perimeter (in kilometers) or to choose from default values (e.g. 35 km for apprentices, 50 km for “normal” workers). This way, the job seeker can limit its search within a certain radius. Data on the search perimeter reported in the search profiles are available^6^ from 2017:5 onwards. Since then, there have been a total of 140 million single entries ranging from 1 to 900 kilometres. This information is exploited in order to measure the average monthly search perimeter (*SP*) and to empirically check how it changes alongside the economic or labour market cycle.

From a data quality perspective, the search intensities introduced in this paper have several advantages. They are based on big data directly capturing online activity. Thus, they can build on large samples and do not have to rely on survey data or on counting actual applications. Furthermore, they are available without any publication lag. This makes them especially valuable during times of big turmoil such as the COVID-19 crisis, while lagged standard data make it difficult to assess the current situation of the economy and the labour market. The novel data open up a new range of possibilities. Policy makers might use this high frequency measure to adjust specific policies more quickly. It could also help nowcasting the economy more accurately, which is important for researchers in that area but also for the government for budget reasons.

This being said, the novel data also have their limitations. Netmind provides only aggregate data that cannot be merged to individual or administrative data sets. It is possible to distinguish between visits and activated visits and whether the job seekers’ or the firms’ part of the online job exchange was accessed. However, Netmind does not provide access to interesting other information such as the free texts entered by the job seekers in the search masks, e.g. on the desired occupation. Furthermore, the job seekers of the online job exchange might not be representative for all job seekers since on-the-job searchers could use other search channels, too. However, for an important subgroup of the job seekers (the unemployed), the FEA is the (legally required) contact point and a central intermediary. In view of the matching function that will be discussed in Sect. 3, the data at hand should be a very good proxy for search intensity of the unemployed. Consequently, it can serve as a valuable additional ingredient for the matching function (which does not model on-the-job search but the part of matches that stem from an outflow from unemployment).

**Fig. 1:**
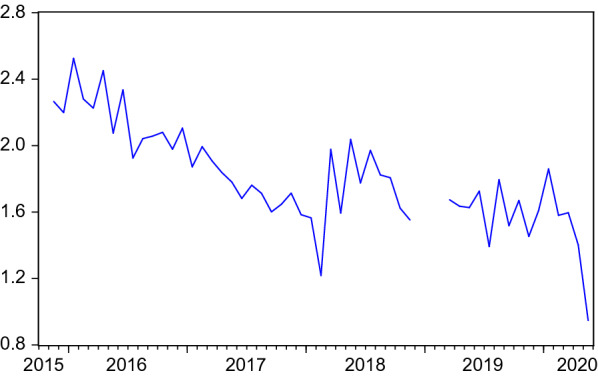
Search intensity of job seekers. Activated visits on the FEA’s job exchange website by job seekers per working day normalised by number of unemployed. Structural breaks after periods of missing data are eliminated by level shift dummies in ARMA models. Seasonally adjusted data

**Fig. 2:**
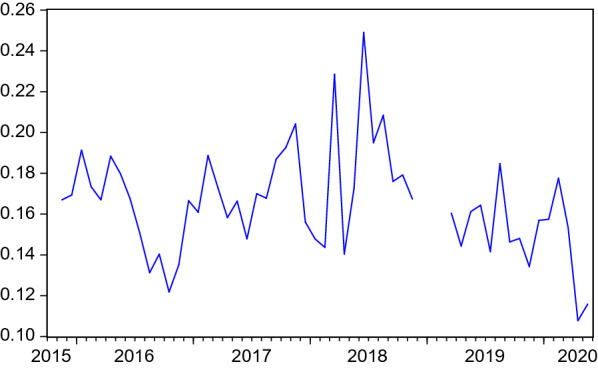
Search intensity of firms. Activated visits on the FEA’s job exchange website by firms per working day normalised by number of vacancies. Structural breaks after periods of missing data are eliminated by level shift dummies in ARMA models. Seasonally adjusted data

**Fig. 3:**
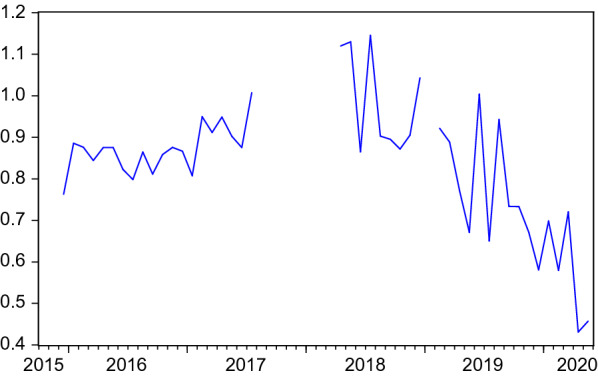
Placement intensity of employment agencies. Activated visits (performing genuine placement activities) on the VerBIS platform by employment agents per working day normalised by the sum of unemployed and vacancies. Structural breaks after periods of missing data are eliminated by level shift dummies in ARMA models. Seasonally adjusted data

Figures 1, 2 and 3 depict the development of the resulting monthly search and placement intensities since they are available (2015:11). Table 1 shows the respective summary statistics. All three intensity measures show relevant variation during the sample. In the COVID-19 crisis, they experience a dramatic drop, reflecting the firms’ reluctance to hire and difficulties for employment agencies and job centers in pursuing their placement tasks under corona conditions and high priority attached to short-time work. Job seekers’ search intensity decreased, too, both due to a decreasing nominator (activated visits) but also due to an increasing denominator (unemployment).

**Table 1:** Summary statistics of search intensities and search perimeter

	$I^{JS}$	$I^{F}$	$I^{EA}$	*SP*
Mean	1.8089	0.1651	0.8389	44.1953
Median	1.7783	0.1664	0.8716	44.2159
Maximum	2.5255	0.2490	1.1456	44.7406
Minimum	0.9472	0.1077	0.4310	43.5653
Std. dev.	0.3055	0.0261	0.1538	0.3307
Observations	52	52	45	32

$I^{JS}$, $I^{F}$, $I^{EA}$: Intensities are measured as activated visits by job seekers / firms / employment agencies per working day per number of unemployed / vacancies / unemployed+vacancies. *SP*: Average search perimeter of job seekers in kilometers

**Fig. 4:**
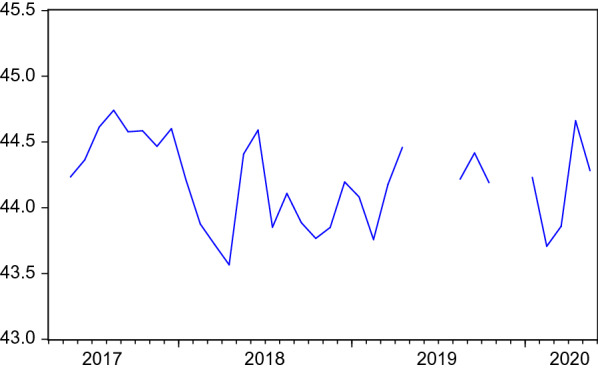
Search perimeter of job seekers. Average search perimeter of job seekers’ search profiles on the FEA’s job exchange website. Structural breaks after periods of missing data are eliminated by level shift dummies in ARMA models. No seasonal pattern was found in the data

Figure 4 and the last column of Table 1 show the development and summary statistics of the job seekers’ search perimeter. It moves within a rather limited range of 1.2 km. This is mainly due to the measure being a monthly *average* among all entries and needs to be kept in mind when interpreting the regression results in Sect. 4.2 with respect to economic relevance.

## Theoretical considerations

Search and matching theory (e.g. [19, 21]) provides guidance for what can be expected regarding the cyclical behaviour of search activities. It states that vacancies (*V*) and unemployed (*U*) form matches (*H* for hirings) through a Cobb-Douglas production function. After log-linearisation, the matching function reads

1 $$\begin{aligned} ln(H_{t}) = \mu _{t} + {\alpha }ln(V_{t-1}) + ({1-\alpha })ln(U_{t-1}), \end{aligned}$$

where $\alpha$ and $(1-\alpha )$ are the elasticities of new matches with respect to vacancies and unemployed, respectively, under the assumption of constant returns to scale. Matching efficiency $\mu _t$ represents the productivity measure of this function. It depends on determinants such as the institutional quality of employment services, search intensity, willingness to take up work, or mismatch (compare [3, 12, 16]). Since time variation in matching efficiency can be substantial (e.g. [12, 23]), matching efficiency is allowed to vary over time. Subtracting $ln(U_{t-1}$ from both sides of the equation yields

2 $$\begin{aligned} ln(jfr_{t}) = \mu _{t} + {\alpha }ln(\theta _{t-1}), \end{aligned}$$

where *jfr* denotes the job finding rate and $\theta =V/U$ labour market tightness.

This theoretical framework has several implications for the cyclical behaviour of search intensity. From the firms’ perspective, an upswing is—ceteris paribus—connected to decreasing unemployment and hence a lower level of hirings (Eq. 1). However, the firms can react by posting more vacancies and increasing search effort (Eq. 2) in order to obtain the same level of hirings. In fact, there is evidence in the literature supporting this reasoning. Davis et al. [3], for instance, find procyclical recruitment intensity. Employers increase their hiring efforts in stronger - and thus tighter - labour markets in order to fill their positions.

From the job seekers’ perspective, applying the same reasoning leads to counter-cyclical search behaviour. A stronger labour market with lower unemployment levels leads—ceteris paribus—to higher job offer arrival rates (see, e.g. [19]), requiring less search effort from the job seeker to obtain the same level of hirings. In a similar reasoning, DeLoach and Kurt [5] argue that search effort can be countercyclical because it is intensified in downturns to prevent declines in household wealth. A procyclical search effort on the firms’ side and countercyclical search intensity on the job seekers’ side is in line with the compensation argument: Both market sides can compensate for a tighter (firms) or worse (job seekers) labour market via increased search efforts. This would also apply for the search perimeter since job seekers’s willingness to compromise probably increases as the labour market situation worsens. However, one could also think of a mechanism where lower chances to receive a job offer in times of labour market slackness can discourage search [5]. Then, job seekers’ search intensity (and probably also search perimeter) would be procyclical if the discouragement mechanism dominates. In this context, it is possible that the perceived severity and permanence of a downturn could influence the relative importance of the two competing channels. If a crisis is not being perceived as transitory, it could well be that the discouragement effect dominates the compensation effect.

The behaviour of the third actor, the employment agency, can also vary over time, although it is not clear a priori whether it follows the cycle of the demand or supply side, or none at all. One could think of procyclical placement behaviour if a tighter labour market requires the agency to more intensively support firms in their search for suitable candidates. By contrast, the agency might intensify support for unemployed if the economic conditions worsen. It is conceivable, for example, that the job search could be made more comprehensive beyond the standard or that the placement officer to job seeker ratio could be improved. Hence, the cyclical behaviour of the search and placement activities is an empirical question that will be answered in the following.

## Cyclicality and seasonality of search and placement activities

### Scatter plots on cyclicality

To get a first impression, this subsection analyses the cyclical behaviour of search and placement intensity via scatter plots.

**Fig. 5:**
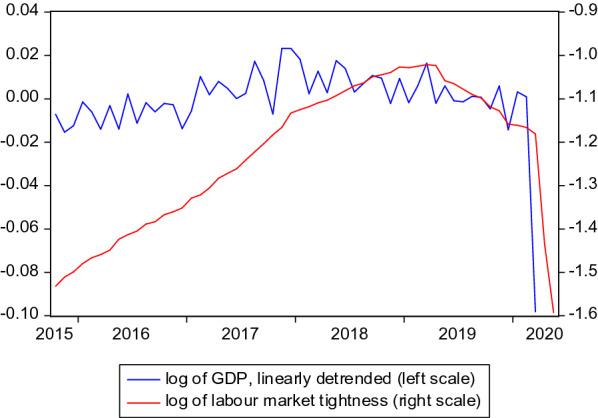
GDP and labour market tightness. GDP: Imputed data [6] using quarterly GDP as anchor variable and monthly industrial production as auxiliary variable. Source: Federal Statistical Office. Labour market tightness: Number of vacancies divided by number of unemployed. Source: Federal Employment Agency. Seasonally adjusted data

First, it needs to be addressed whether “cycle” means the business cycle or the labour market cycle. Beyond the fact that the latter usually lags the former, Klinger and Weber [13], for instance, document a sizeable decoupling between business cycle and the labour market in Germany, especially so during the last decade. To cover both, gross domestic product (*GDP*) and labour market tightness ($\theta$), defined as number of vacancies divided by the number of unemployed, are used. Calendar- and seasonally adjusted GDP (index: 2015=100) was taken from the Federal Statistical Office (FSO). It is only available at a quarterly frequency, which is why the imputation algorithm by Denton [6] was implemented using industrial production (also from the FSO) as auxiliary variable in order to generate a monthly GDP time series. Figure 5 shows that there was a rather stable upswing in GDP until the end of 2017 before the downturn in 2018 and 2019. Labour market tightness grew strongly until the end of 2017, followed by a period of slower growth until mid of 2019, after which it steadily decreased. Both variables dropped severely due to the COVID-19 crisis. All in all, both variables experienced upswing and downturn during the sample even before COVID-19, which allows to investigate the cyclical patterns of the search and placement intensities.

**Fig. 6:**
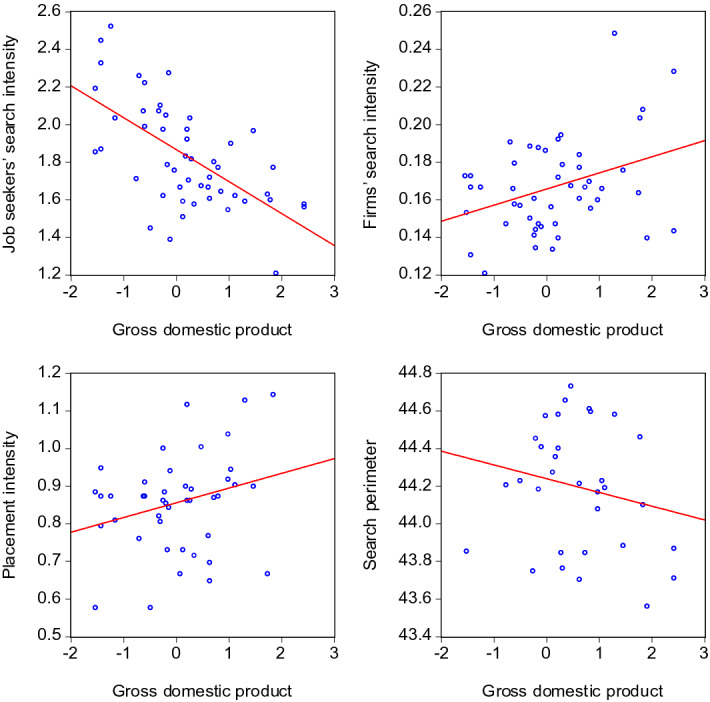
Scatter plots of search intensities and GDP. Gross domestic product: Linearly detrended data (i.e. a linear trend was subtracted from the original data). Search activity variables: The lags of *GDP* yielding the strongest correlation in the regressions of Table 2 were used in the scatter plots: $I^{JS}_{t}$ and $GDP_{t-1}$, $I^{F}_{t}$ and $GDP_{t-3}$, $I^{EA}_{t}$ and $GDP_{t-2}$, $SP_{t}$ and $GDP_{t-3}$. The red lines visualise the fitted linear relationship stemming from OLS regressions

**Fig. 7:**
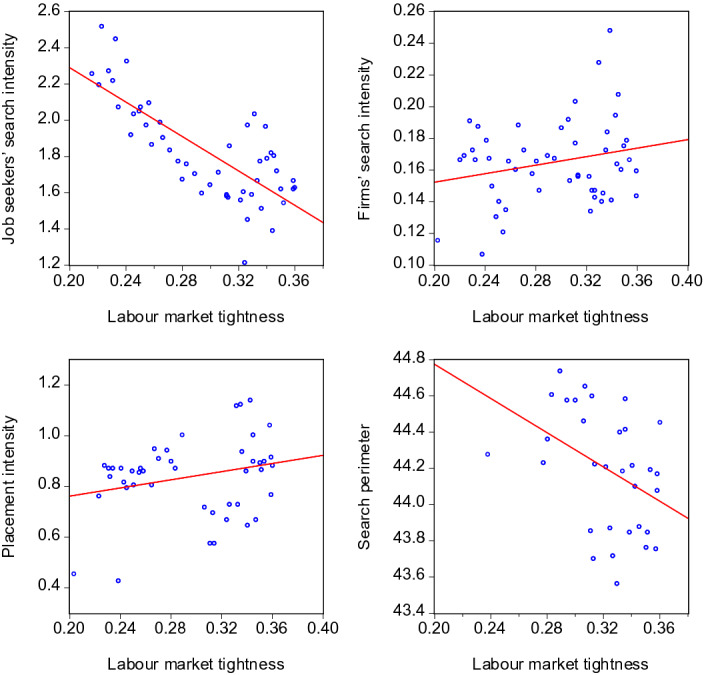
Scatter plots of search intensities and labour market tightness. Labour market tightness: Number of vacancies divided by number of unemployed. The lags of $\theta$ yielding the strongest correlation in the regressions of Table 3 were used in the scatter plots: $I^{JS}_{t}$ and $\theta _{t-1}$, $I^{F}_{t}$ and $\theta _{t}$, $I^{EA}_{t}$ and $\theta _{t-2}$, $SP_{t}$ and $\theta _{t-1}$

A visual impression of the behaviour of the search and placement intensities in the course of a business or labour market cycle can be obtained by contrasting them with GDP and tightness in scatter plots. This provides a first idea about the pro- or countercyclical nature of the search activities on the labour market. Figures 6 and 7 show the pairwise relationships for GDP and labour market tightness, respectively. All scatter plots contain data until 2020:3 and hence exclude the extreme months of the COVID-19 crisis.

Both the placement intensity of employment agencies and the firms’ search intensity seem to move pro-cyclically. The opposite holds for the labour supply side: Job seekers’ search intensity and their search perimeter show signs of counter-cyclical movements.

**Fig. 8:**
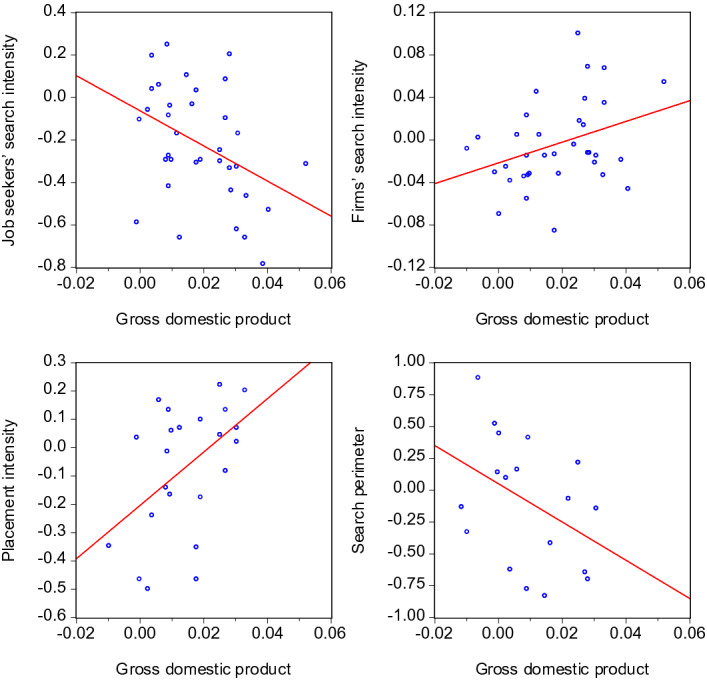
Scatter plots of search intensities and GDP—annual differences. The notes of Table 6 apply, except that annual *differences* of *GDP*, $I^{JS}$, $I^{F}$, $I^{EA}$, and $SP$ have been used

**Fig. 9:**
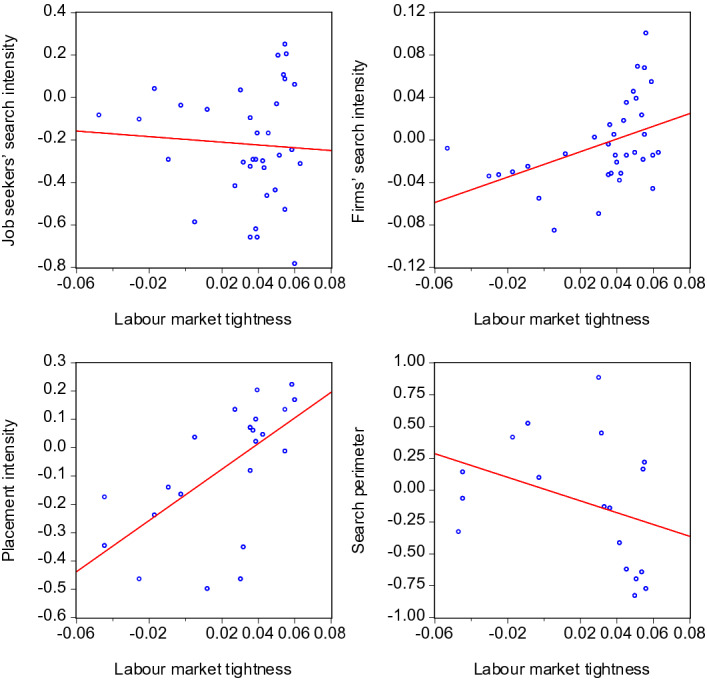
Scatter plots of search intensities and labour market tightness—annual differences. The notes of Table 7 apply, except that annual *differences* of $\theta$, $I^{JS}$, $I^{F}$, $I^{EA}$, and $SP$ have been used

To the extent the variables are subject to persistence, there is a risk that the respective scatter plots might display pro- or countercyclicality due to spurious correlations. However, the following scatter plots based on the annual *differences* of the variables show that this risk does not materialize here. Differencing, i.e. subtracting lagged values, removes potential stochastic as well as deterministic trends from the time series and hence is a suitable method to deal with persistent time series. Figures 8 and 9 show that in all cases, the scatter plots look similar to those of Figures 6 and 7. Hence, the visual impressions regarding pro- or countercyclicality are confirmed for both the level and the differenced variables.

### Regression analysis

The scatter plots gave a first impression. The following steps involve a more formal analysis in which also information about significance and lag structure of the cyclical relationships can be obtained. While so far it remained unclear how long it takes for the cycle to materialise in the search activities on the labour market, regression analysis is suited to address this question. Including contemporaneous as well as lagged cycle variables lets the data speak about whether search activity reacts immediately or after a delay to cyclical movements. For each possible combination of search activity and cycle variable, the following regression equation is estimated:

3 $$\begin{aligned} ln(y_{t}) = \alpha + \sum _{i=0}^{p}\beta _{i}ln(x_{t-i}) + \gamma t + \epsilon _{t} , \end{aligned}$$

where $y\in (I^{JS}, I^{F}, I^{EA}, SP)$, $x\in (GDP, \theta )$, *p* is the lag length, and $\epsilon$ is a normally-distributed error term. Equation (3) controls for a linear trend ($\gamma t$). This way, $\beta _{0}$ to $\beta _{p}$ capture the *cyclical* effects and do not pick up correlation stemming from linear trends in the variables. As in the scatter plots, the estimation period does not cover the COVID-19 crisis months 2020:4 and 2020:5 due to their extreme values.

A further matter of interest are data properties such as the persistence of variables in the given sample period. The scatter plots already gave a first impression that this might not be a decisive issue here. Indeed, Augmented Dickey-Fuller (ADF) tests confirm that GDP does not show the persistence usually found in longer samples: They find that GDP behaved as trendstationary variable during the sample at hand. The null hypothesis of GDP having a unit root is rejected at the 1% significance level in an ADF test with a constant and a deterministic trend as exogenous variables. Nonetheless, regressions on the annual *growth rate* of GDP were conducted as robustness check in Sect. 4.4.

Equation (3) is estimated using ordinary least squares (OLS) with heteroscedasticity- and autocorrelation-robust standard errors. All variables appear in logs. As baseline, a lag length of $p=3$ is chosen to allow delayed effects up to a quarter (i.e. three months). A robustness check on the lag length is presented in Sect. 4.4. Tables 2 and 3 show the results.

**Table 2:** Regression results: search intensities and GDP

	Dependent variable							
	$I^{JS}$		$I^{F}$		$I^{EA}$		*SP*	
Constant	39.23***	(14.05)	− $$27.31{*}$$	(14.39)	− $$77.84{***}$$	(13.53)	$$7.18{***}$$	(0.75)
$GDP_{t}$	0.39	(0.81)	0.27	(1.14)	0.15	(1.29)	0.09**	(0.04)
$GDP_{t-1}$	− 5.55***	(1.08)	− 0.63	(2.35)	3.46**	(1.74)	− 0.13	(0.12)
$GDP_{t-2}$	− 1.65	(1.76)	0.07	(1.53)	7.22*	(4.03)	− 0.26**	(0.11)
$GDP_{t-3}$	− 1.52	(0.99)	5.82***	(0.74)	6.03**	(3.03)	− 0.43***	(0.14)
trend	0.45	(0.42)	− 0.81**	(0.33)	− 2.69***	(0.45)	0.04**	(0.02)
R-squared	0.6684		0.1505		0.5104		0.4409	

Estimated effects from OLS regressions with heteroscedasticity- and autocorrelation-robust standard errors (in parentheses). *, **, *** denote significance at the 10, 5, 1% level, respectively. $I^{JS}$, $I^{F}$, $I^{EA}$: Search intensity of job seekers/firms/employment agencies. *SP*: Average search perimeter of job seekers in kilometers. All variables enter the regressions in logarithms

**Table 3:** Regression results: Search activities and tightness

	Dependent variable							
	$I^{JS}$		$I^{F}$		$I^{EA}$		*SP*	
Constant	0.74	(0.51)	− $$1.41{***}$$	(0.46)	$$1.38{***}$$	(0.24)	$$3.76{***}$$	(0.06)
$$\theta _{t}$$	− 0.67	(1.33)	$$2.75{**}$$	(1.38)	$$5.29{***}$$	(1.36)	0.22	(0.29)
$$\theta _{t-1}$$	− $$2.28{*}$$	(1.28)	− 0.74	(2.01)	− $$6.81{*}$$	(3.84)	− $$0.32{**}$$	(0.12)
$\theta _{t-2}$	0.50	(3.07)	− 2.19	(3.26)	$5.79{*}$	(2.98)	− 0.04	(0.25)
$$\theta _{t-3}$$	2.32	(3.67)	0.50	(2.74)	− $$3.20{**}$$	(1.58)	0.11	(0.13)
trend	$$0.94{**}$$	(0.41)	− 0.08	(0.38)	− $$0.96{***}$$	(0.16)	− 0.01	(0.06)
R-squared	0.6050		0.1214		0.6863		0.2784	

Estimated effects from OLS regressions with heteroscedasticity- and autocorrelation-robust standard errors (in parentheses). *, **, *** denote significance at the 10, 5, 1% level, respectively. $I^{JS}$, $I^{F}$, $I^{EA}$: Search intensity of job seekers/firms/employment agencies. *SP*: Average search perimeter of job seekers in kilometers. $\theta$: labour market tightness. All variables enter the regressions in logarithms

In general, they confirm the visual impression obtained by the scatter plots: $I^{F}$ and $I^{EA}$ move pro-cyclically while $I^{JS}$ and *SP* move counter-cyclically. Furthermore, these relationships prove to be significant in any of the regressions for at least one lag, which is remarkable given the limited number of observations.

Although I would recommend not to over-interpret the lag structure at this early stage, one result seems evident: On average, the reactions to labour market cycle movements are quicker than to business cycle movements. For instance, a tighter labour market materialises instantaneously in higher search efforts of firms (second column of Table 3) while it needs a while in case of *GDP*-changes (second column of Table 2). While surprising at first glance, it could indeed be rational for firms not to react immediately to (potentially short-lived) fluctuations but instead to wait until an economic upswing or downturn proves to be stable before making any decisions with respect to their search behaviour. Furthermore, the labour market itself lags the real economy.

The significant effects are also relevant in size, although to different extents: For instance, a positive 1% *GDP* change increases $I^{F}$ and decreases $I^{JS}$ by 5 to 6%, while it increases $I^{EA}$ by almost 17% after 3 months. In case of $\theta$, the effects are about half the size, which is compensated by the fact that during the sample, the variation of tightness is much higher than that of GDP.

With respect to placement intensity, the changing signs (positive for lags of 0 and 2, negative for lags 1 and 3) indicate that the *growth rate* of tightness matters for placement intensity.^7^ Indeed, when estimating the third column of Table 3 with *differenced* log of *theta* instead of the level, the signs do not “jump” anymore, and $\beta _{0}$ and $\beta _{2}$ are highly significant with estimated effects of 6 to 7%.

At first glance, the effects seem to be less relevant in case of the search perimeter. They are much smaller, with the sum of the effects ranging between − 0.3 and − 0.5% after 3 months (fourth column). However, the standard deviation of *SP* amounts to only 0.33 km (or 0.75% in terms of its average). Hence, put into context, the estimated effects are relevant after all.

Overall, the explanatory power of the trend and cycle variables varies from 12 to 69%. While the search intensity of firms is obviously influenced a lot by factors beyond the aggregate business or labour market cycle, two thirds of the variation in job seekers’ search intensity can be explained by the business cycle (the trend is irrelevant here).

In a more general setting, regression analysis allows both *GDP* and $\theta$ to play a role. By including both variables in the same equation, it is possible to know whether there is a dominant cycle that influences search and placement activities. Table 4 shows the results.

In case of the job seekers’ search intensity, the results show that the business cycle dominates the labour market cycle. The dependence of the firms’ search intensity on the cycle variables remains rather unchanged when both *GDP* and $\theta$ are included in the regression. It is still contemporaneous labour market tightness and the 3-month lag of GDP that exert the procyclical effects. In addition, also the size of the estimated effects barely change. In case of placement intensity, it is the labour market cycle that dominates the business cycle. The coefficients of $\theta$ do not change much, which means also the more complex lag structure found above remains. Both *GDP* and $\theta$ remain significant factors for the search perimeter of the job seekers even when both are added. Also the lag structure remains unchanged compared to Tables 2 and 3.

To sum up, for job seekers and firms, the economic cycle seems to be the more relevant factor, while employment agencies are mainly driven by the labour market cycle.

**Table 4:** Regression results including both GDP and tightness

	Dependent variable							
	$I^{JS}$		$I^{F}$		$I^{EA}$		*SP*	
Constant	$$63.08{***}$$	(16.89)	6.22	(25.23)	− 5.45	(20.72)	$$6.66{***}$$	(1.82)
$GDP_{t}$	− 0.26	(0.66)	− 0.48	(0.98)	− 1.50	(1.24)	0.10	(0.08)
$$GDP_{t-1}$$	− $$6.78{***}$$	(2.10)	− 4.47	(3.16)	− 0.23	(2.11)	− 0.06	(0.20)
$GDP_{t-2}$	− $3.26{*}$	(1.69)	− 1.96	(2.38)	0.80	(3.64)	− $0.24{*}$	(0.13)
$$GDP_{t-3}$$	− $$2.98{**}$$	(1.42)	$$5.21{***}$$	(1.94)	2.44	(2.54)	− $$0.42{***}$$	(0.14)
$$\theta _{t}$$	0.06	(1.35)	$$2.66{*}$$	(1.34)	$$5.49{***}$$	(1.30)	0.10	(0.22)
$$\theta _{t-1}$$	− 0.53	(1.51)	1.52	(1.53)	− $$7.38{**}$$	(3.49)	− $$0.22{*}$$	(0.11)
$\theta _{t-2}$	1.31	(3.49)	− 3.21	(3.14)	$5.22{*}$	(3.03)	0.16	(0.26)
$\theta _{t-3}$	− 0.19	(3.35)	− 0.74	(2.89)	− 2.19	(1.85)	− 0.05	(0.29)
trend	0.51	(0.58)	0.41	(0.69)	− $$1.35{**}$$	(0.60)	0.04	(0.07)
R-squared	0.6893		0.2330		0.7051		0.4636	

Estimated effects from OLS regressions with heteroscedasticity- and autocorrelation-robust standard errors (in parentheses). *, **, *** denote significance at the 10, 5, 1% level, respectively. $I^{JS}$, $I^{F}$, $I^{EA}$: Search intensity of job seekers/firms/employment agencies. *SP*: Average search perimeter of job seekers in kilometers. $\theta$: labour market tightness. All variables enter the regressions in logarithms

### Controlling for compositional effects

While the results so far reveal cyclical patterns of the search activities, one has to be careful in interpreting these findings in terms of changing search *behaviour*. Beyond the search behaviour, also the composition of the searchers could change during an upswing or downturn and hence influence the observed search intensity. For instance, the composition of the monthly inflow into unemployment could (partly or fully) explain the cyclical patterns of the search activities, not only of job seekers but also of firms and placement agents. To account for such potential compositional effects, this subsection adds several control variables to all regression equations.

The set of monthly available variables capturing relevant characteristics of job seekers comprises the shares of high-education (college degree), low-education (neither vocational training nor high school degree), older ($>55$ years of age), younger ($<=25$ years of age), female, and of foreign people among total inflow into unemployment. The shares are taken from the FEO’s statistics. Table 5 shows the results.

**Table 5:** Regression results: controlling for compositional effects

	Dependent variable							
	$I^{JS}$		$I^{F}$		$I^{EA}$		*SP*	
Constant	$$78.01{***}$$	(27.87)	$$50.75{*}$$	(28.18)	− 8.69	(24.00)	$$3.55{***}$$	(1.06)
$GDP_{t}$	− 0.40	(1.01)	− 1.16	(1.27)	− 1.66	(0.97)	0.12	(0.08)
$$GDP_{t-1}$$	− $$7.16{**}$$	(2.90)	− 5.54	(3.95)	0.47	(2.62)	0.02	(0.12)
$GDP_{t-2}$	− 3.53	(2.25)	− 3.48	(2.62)	3.05	(3.65)	− 0.02	(0.12)
$$GDP_{t-3}$$	− $$3.31{*}$$	(1.64)	$$4.09{*}$$	(2.33)	1.46	(2.48)	− $$0.24{**}$$	(0.12)
$$\theta _{t}$$	0.33	(1.54)	− 0.99	(1.63)	$$7.29{***}$$	(2.35)	$$0.36{***}$$	(0.11)
$$\theta _{t-1}$$	0.02	(1.74)	$$5.06{**}$$	(2.20)	− $$9.74{*}$$	(4.01)	− $$0.34{*}$$	(0.18)
$\theta _{t-2}$	0.60	(3.87)	− 2.55	(3.59)	5.24	(3.72)	0.00	(0.17)
$\theta _{t-3}$	− 0.70	(3.28)	− 0.82	(2.86)	− 1.63	(3.00)	− 0.02	(0.14)
trend	− 0.38	(1.02)	0.06	(1.70)	− $$2.65{**}$$	(0.97)	0.14	(0.12)
R-squared	0.7668		0.3986		0.7968		0.6271	

The notes of Table 4 apply. The control variables comprise the shares of high-education (college degree), low-education (neither vocational training nor high school degree), older ($>55$ years of age), younger (≤ 25 years of age), female, and of foreign people among total inflow into unemployment

With respect to the job seekers’ search intensity, the control variables are able to explain an additional share of the variation in search intensity (The R-squared increases by 8% points). Including them does not lead to decreasing cyclical effects, though. The effects of job seekers’ search intensity with respect to *GDP* remain basically unchanged. However, statistical significance is slightly weaker when control variables are included.

In case of the firms’ search intensity, adding the control variables increases the R-squared by a substantial amount (almost 17% points). However, the procyclical movement found so far does not disappear. Both *GDP* and $\theta$ remain significant factors, although the contemporaneous effect of $\theta$ becomes insignificant and is replaced by the first lag instead.

Also for the relationship between placement intensity and cycle, the control variables do not play a game-changing role. Although adding them increases the R-squared by approximately 9% points, the estimated cyclical coefficients do not change much. Again, their statistical significance is slightly weaker when control variables are included.

In case of the search perimeter, adding the control variables increases the R-squared by 16% points. The countercyclical behaviour with respect to labour market tightness disappears although it remains intact with respect to the business cycle. The results on the search perimeter, however, are based on 30 observations only, so estimating 16 parameters might be a challenge that is just a bit too high. Thus, probably a bit more time is warranted in order to collect longer time series before eventually answering this question for the search perimeter.

To sum up, there is some evidence that observable compositional effects are able to explain a part of the development of search and placement activities. However, they do not substantially weaken the findings regarding the cyclical behaviour. Of course, it is still possible that there are unobservable compositional effects at play. For instance, the relation of unemployed searchers versus on-the-job searchers could change during an upswing or downturn. Similarly, it is possible that job seekers and firms change their preferred search channel during a business cycle, which could potentially change the composition of job seekers or firms using the FEA’s online job exchange. Although the aggregate search activity data at hand do not allow to look into the details of *who* accesses the websites but only *how often* the websites are accessed, there is little evidence that changing search channel preferences on the firms’ side play a decisive role during the sample period. According to the job vacancy survey of the Institute for Employment Research (see, e.g. [17]), the FEA’s online job exchange was mentioned as relevant search channel in one third of the cases in every single year since 2015,^8^ which does not leave much room for major compositional effects.

Furthermore, one could think of other potential control variables. For instance, the reservation wage could be a relevant factor for the job seekers' search behaviour. However, the reservation wage cannot be observed directly but at most be modelled as a function of labour market tightness, the structure of the unemployed (both of which are accounted for in the regressions), or of the net replacement rate of the unemployed. With regards to the latter, annual OECD data^9^ show that the net replacement rate basically remained unchanged since 2015 at 59%, so again no major effects can be expected here.

Against the background of the theoretical reasoning in Sect. 3, the results indicate that job seekers and firms seem to compensate difficulties on their respective market sides with increased search on the labour market. Job seekers search more and widen their search perimeter in times of weaker economy and labour market while firms search more in times of stronger economy and labour market. Since the employment agencies are the intermediary between both market sides, their cyclical behaviour is ambiguous from a theoretical point of view. The empirical results indicate that—like firms’ search intensity—placement intensity increases when the labour market becomes tighter. A potential explanation could be that in times of tighter labour markets there are shifts within the FEA away from the placement of unemployed towards the employer service (“Arbeitgeberservice”, a department of the FEA supporting firms in finding employees).^10^

### Robustness checks

To check robustness of the results, richer specifications are estimated using 6 instead of 3 lags of *GDP* or $\theta$ to allow for delayed effects up to half a year. The choice of the lag length is supported by statistical tests and information criteria. For instance, in no case do the Schwarz criterion and the Akaike information criterion recommend using a lag length of more than 6 months. Tests based on the Ljung-Box Q-statistics reveal that the null hypothesis of no serial correlation in the residuals is not rejected at the 5 percent significance level in case of p = 3, and even at the 10 percent level in case of p = 6. Table 6 shows the results for p = 6.

**Table 6:** Robustness checks on lag length

	Dependent variable							
	$I^{JS}$		$I^{F}$		$I^{EA}$		*SP*	
GDP								
Constant	$$26.23{**}$$	(12.69)	− $$46.00{***}$$	(12.98)	− $$95.16{***}$$	(5.04)	$$8.00{***}$$	(0.93)
$$GDP_{t}$$	0.01	(0.61)	− 0.45	(0.98)	− 1.12	(1.23)	$$0.07{**}$$	(0.03)
$$GDP_{t-1}$$	− $$6.80{***}$$	(1.69)	− 2.52	(1.89)	− 0.85	(3.19)	− 0.16	(0.15)
$$GDP_{t-2}$$	− $$2.51{**}$$	(1.02)	− 1.18	(1.43)	3.51	(3.31)	− $$0.34{**}$$	(0.13)
$$GDP_{t-3}$$	− 1.85	(1.72)	$$5.40{***}$$	(1.16)	$$5.19{*}$$	(3.07)	− $$0.48{***}$$	(0.11)
$$GDP_{t-4}$$	2.40	(2.63)	0.86	(1.01)	$$4.53{**}$$	(2.19)	− $$0.24{*}$$	(0.13)
$$GDP_{t-5}$$	0.18	(1.35)	1.64	(3.26)	$$3.39{**}$$	(1.42)	− 0.03	(0.16)
$$GDP_{t-6}$$	3.05	(2.08)	$$5.83{***}$$	(1.80)	$$5.99{**}$$	(2.83)	$$0.27{***}$$	(0.08)
Trend	0.04	(0.40)	− $$1.42{***}$$	(0.32)	− $$3.42{***}$$	(0.17)	$$0.04{*}$$	(0.02)
Tightness								
Constant	$$1.74{*}$$	(0.89)	− 0.53	(1.11)	$$1.99{***}$$	(0.64)	$$3.87{***}$$	(0.05)
$$\theta _{t}$$	0.37	(1.29)	$$3.64{**}$$	(1.43)	$$6.33{***}$$	(0.84)	$$0.42{**}$$	(0.20)
$$\theta _{t-1}$$	− $$2.42{**}$$	(1.17)	− 0.68	(1.60)	− $$8.04{**}$$	(3.09)	− $$0.39{*}$$	(0.22)
$\theta _{t-2}$	− 0.51	(3.51)	− 2.91	(3.27)	$5.24{*}$	(2.85)	− 0.09	(0.26)
$$\theta _{t-3}$$	− 0.52	(3.50)	− 0.38	(3.98)	− $$6.44{***}$$	(2.24)	− 0.25	(0.18)
$$\theta _{t-4}$$	2.11	(2.30)	-1.27	(4.81)	$$6.59{***}$$	(1.85)	0.01	(0.15)
$$\theta _{t-5}$$	0.33	(3.33)	0.71	(2.69)	− $$3.48{**}$$	(1.49)	0.13	(0.27)
$\theta _{t-6}$	1.12	(1.09)	1.76	(2.69)	1.23	(2.61)	0.21	(0.20)
Trend	− $$1.73{**}$$	(0.68)	− 0.69	(0.82)	− $$1.49{**}$$	(0.61)	− $$0.11{***}$$	(0.04)

Estimated effects from OLS regressions with heteroscedasticity- and autocorrelation-robust standard errors (in parentheses). *, **, *** denote significance at the 10, 5, 1% level, respectively. $I^{JS}$, $I^{F}$, $I^{EA}$: Search intensity of job seekers/firms/employment agencies. *SP*: Average search perimeter of job seekers in kilometers. $\theta$: labour market tightness

They confirm the general results of Tables 2 and 3 on the cyclical behaviour of the search and placement activities. In many cases, also the same lags of the cycle variables are significant—and they are similar in size. For $I^{F}$, the 6th lag of *GDP* is significant, too, substantially increasing its procyclical response. Thus, the smaller model with 3 lags can be considered conservative. For $I^{EA}$, the bigger model prefers more delayed reactions to GDP-changes, and the sum of the effects is a bit higher than in the specification with 3 lags. In case of *SP*, the significant effects of the 4th and 6th lag of *GDP* balance each other out so that the total effect does not change much. However, the countercyclical reaction of *SP* to $\theta$ disappears in the specification with 6 lags, a phenomenon already found when control variables were added.

In a further robustness check, the regressions are performed on the annual *growth rate* of GDP. The pro- or countercyclical patterns as well as the lag structure do not change substantially, though. In case of $I^{JS}$, the biggest effect is still stemming from the first lag of GDP growth with an estimated effect of − 4.79. For $I^{F}$, the highest effect of 3.48 is estimated to occur at the third lag of GDP growth. In case of $I^{EA}$, the second lag of GDP growth exerts the strongest effect with an estimated effect of 4.24. And *SP* reacts strongest to the third lag of GDP growth with an effect of − 0.19. All reported effects are found to be statistically significant.

Finally, the monthly available index of industrial production was used instead of imputed GDP. However, the results do not change substantially. $I^{F}$ and $I^{EA}$ still move pro-cyclically while $I^{JS}$ and *SP* move counter-cyclically.

### The seasonal patterns of search behaviour

**Fig. 10:**
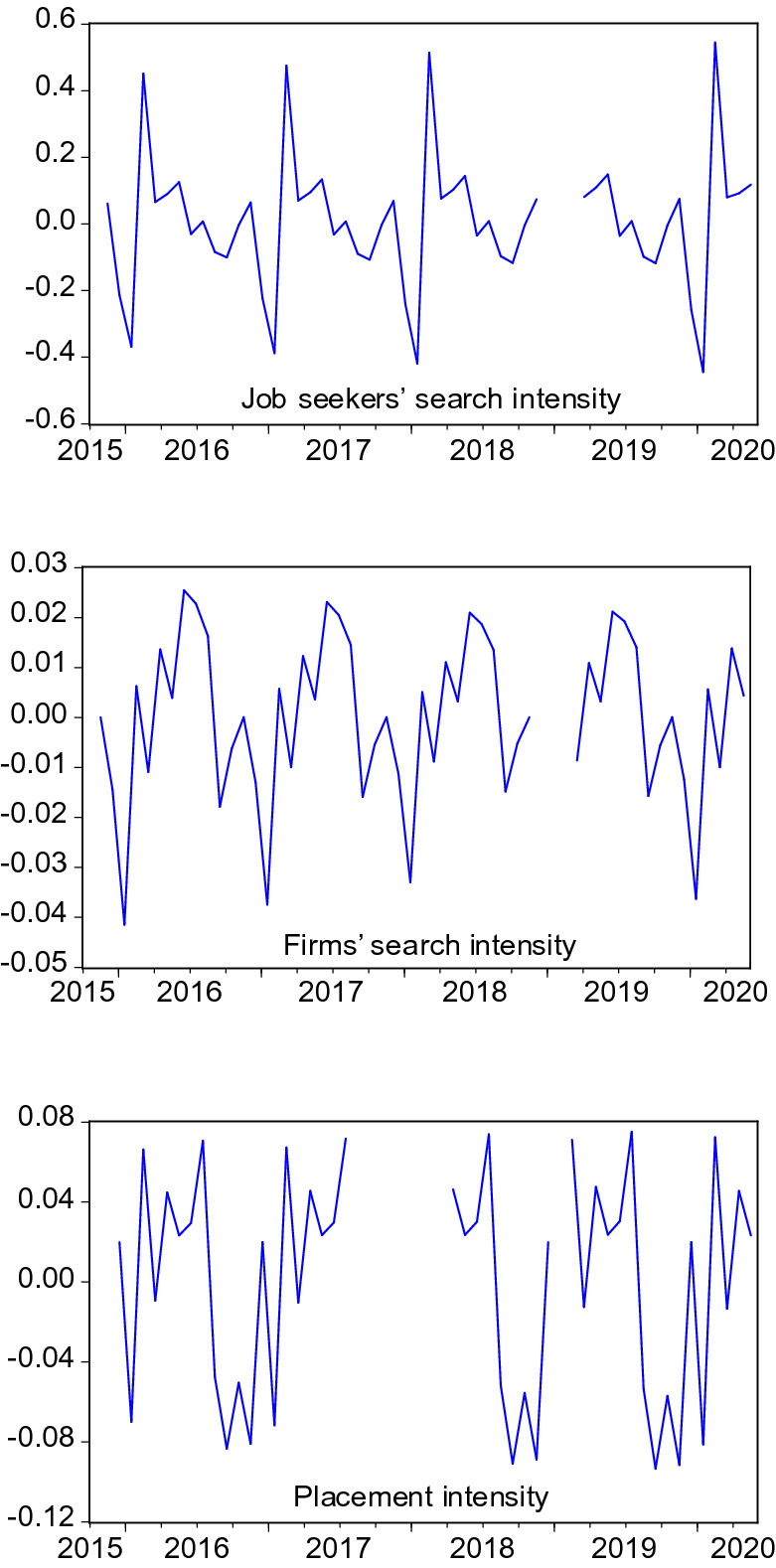
Seasonal patterns of search and placement intensities. The seasonal patterns for search and placement intensities stem from additive X12-ARIMA seasonal adjustment

The data on search and placement activities used until now were seasonally adjusted. Beyond the cyclical movements, also the usual seasonal behaviour in the search and placement intensities could be of interest as it might provide explanations for seasonal patterns in aggregate unemployment or employment. Figure 10 shows the seasonal patterns in the novel data. They are obtained by applying the additive X12-ARIMA seasonal adjustment procedure.

The graphs show how many additional activated visits usually occur in a specific month, beyond the trend-cycle level. For instance, job seekers’ search intensity usually is lowest in January (approximately 0.4 activated visits per unemployed per working day below the trend-cycle level) and highest in February (around 0.5 activated visits above the trend-cycle). Note that the depicted months are not calendar months but months between the counting days (see Sect. 2). Thus, a typical January covers the period from mid of December to mid of January, a typical February goes from mid of January to mid of February, and so forth. Consequently, the period of lowest search intensity on the job seekers’ side usually covers the holiday season of Christmas and New Year’s Eve. Another, less pronounced, local minimum in the seasonal pattern is visible in August and September, most probably due to summer vacation.

The minima of the firms’ search intensity occur in January and September, too. However, the highest efforts can be detected from June to August, most likely due to additional efforts to duly recruit apprentices before the vocational training cycle starts. The firms seem to be supported by the intermediary since July is also the period in which employment agencies undertake the highest placement efforts. The other maximum of placement intensity is in February, matching the maximum of job seekers’ search intensity. And also the minima of placement intensity closely match those of the labour demand and supply sides: They occur in January, September, and November.

Measuring the extent of the seasonal pattern relative to the mean search intensity, the seasonality on the job seekers’ side is found to be a bit higher (ranging from − 25 to + 30% of its mean search intensity) than that on the firms’ side, ranging from − 25 to + 15% (compare also [4] who find that seasonality is much bigger for applications than postings).

All in all, the seasonal patterns seem to be largely influenced by holiday seasons and the vocational training cycle. Since the seasonal patterns on both market sides and the employment agencies largely coincide, they most probably reinforce each others’ effects on the labour market. Consequently, January typically is the month with the highest unemployment rate in the course of a year.

No seasonal pattern could be detected for the search perimeter (neither significant nor relevant in size). This is no surprise given the development shown in Fig. 4. The maxima and minima do not occur at the same months.

## Search and placement during the COVID-19 pandemic

So far, the COVID-19 months were excluded in the analysis. This is justified by the extreme disruption this pandemic has caused, as can be seen in Figs. 1, 2, 3, 4 and 5. Including such extreme values in scatter plots or regression analysis would dominate the results and make it difficult to investigate reactions as they would occur in normal times. At the same time, the long-term consequences of this crisis are not yet clear. It is possible that long-lasting shifts in the economy and the labour market will remain even when the virus is under control.

**Fig. 11:**
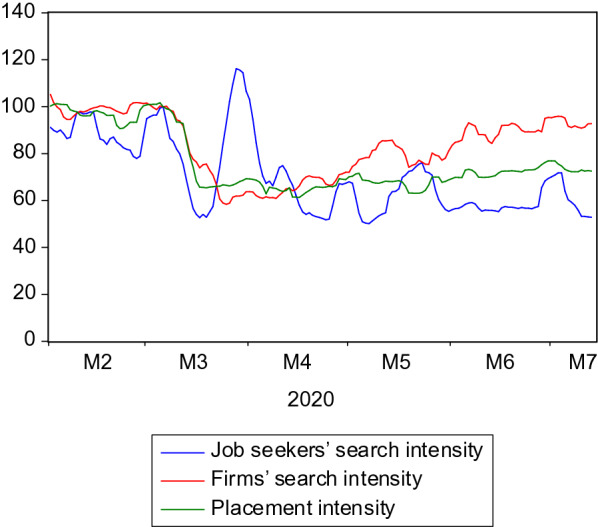
Search and placement during the COVID-19 pandemic. Daily data using a 7-day moving average to eliminate day-of-the-week effects; holiday-adjusted using ARMA models with dummies; index (January 6 2020 = 100)

However, the data at hand allow for a descriptive analysis of search and placement in the COVID-19 pandemic. Since during the first lockdown in March 2020 the news situation changed almost on a daily basis, the fact that the data at hand allow for a daily analysis becomes all the more valuable. Figure 11 shows the daily development of the search and placement intensities of all three agents on the labour market. For scaling purposes, the lines are indexed so that they all start at a value of 100.

Between March 5th and March 25th, the firms’ search intensity dropped substantially by 42%. Until the end of the sample (July 13th), it recovered again, reaching 92.5% of the pre-crisis level. There was also a sharp (− 35%) and even faster (until March 17th) slump of placement intensity, whereas the subsequent recovery proceeded much more slowly. Until the end of the observation period, only 71% of the pre-crisis level had been reached again. Placement intensity declined strongly since employment agents had to prioritize the processing of short-time work requests, in the course of which the placement activity that would be usual in normal times could be carried out only on a considerably reduced scale.

The job seekers’ search intensity displays a more complex development which was hidden in the monthly figures. Within ten days, it initially declined sharply before strongly recovering and even overshooting pre-crisis-levels. However, it quickly declined afterwards to very low levels and has not recovered since. A possible explanation for this development is that an intensified search in the first days after the lockdown was not seen as a priority due to extraordinary challenges to cope with the situation, and the search intensity therefore initially sank. Subsequently, a compensation effect responsible for the usual counter-cyclical search pattern (see Sect. 3) appears to have been dominant until the end of March, while the discouragement effect could have been the dominant driver relatively quickly from April onwards.

## Conclusion

This article introduces innovative online data allowing the instantaneous measurement of search and—for the first time—placement intensity in the labour market in form of online activity. These data are used to estimate their behaviour during the economic and labour market cycle, as well as their usual seasonal patterns. The results show that firms’ and employment agencies’ search and placement intensity displays a pro-cyclical pattern while—at least before the COVID-19 pandemic—job seekers’ search intensity is counter-cyclical.

In the COVID-19 crisis so far, the data reveal that the placement intensity of employment agencies and the firms’ search intensity dropped substantially. Looking at the daily data, the job seekers’ search intensity displays a more complex development. Here, the procyclical drivers seem to eventually dominate during the COVID-19 pandemic, contrary to the counter-cyclical pattern found for normal times. The overall drop in search and placement activities naturally will have adverse impacts on the labour market, especially on the job finding rate [9]. The most critical labour market effects of the crisis may arise not via the separation but via the hiring margin [18]. It is therefore important that the search and placement activities bounce back more quickly after the COVID-19 crisis.

In the future, further data from the BA job exchange could be exploited for scientific use, especially data on search behaviour. Beyond the search perimeter, other entries on the job exchange could be made accessible for research. For instance, the FEA plans a change from Netmind to Matomo, after which information from the search masks such as the desired profession will be saved and could then be accessed for research purposes. Future projects may make it possible to merge the search activity data to administrative data. Then, it could be investigated how characteristics such as qualification, occupation or region affect the search duration. It would also be conceivable to analyze the relationship between the duration of unemployment or the period of time a job vacancy exists and the search intensity.

## Data Availability

The data on online visits of the job exchange platform of the Federal Employment Agency and its internal placement software VerBIS are initially stored in server log files. In an aggregated form, they can be downloaded via the software Netmind. Access is provided by the Federal Employment Agency. The other aggregate data used in this article stem from the Federal Statistical Office (in case of GDP and production index), and from the Statistics Department of the Federal Employment Agency (in case of unemployment, vacancies, and the control variables). They can be downloaded from the respective websites. Alternatively, I can provide the data upon request.

## Footnotes

1 See https://jobboerse.arbeitsagentur.de.

2 It is possible (although not required) for the job seeker to register and log in. The log files are stored irrespective of whether a visitor is logged in on the job exchange website or not.

3 Multiple visits of a specific URL per day by the same visitor are possible and counted as such.

4 The counting days of the FEA’s statistics are published here: https://statistik.arbeitsagentur.de/Navigation/Statistik/Service/Veroeffentlichungskalender/Veroeffentlichungskalender-Nav.html.

5 In addition to job seekers, also firms are supported by employment agencies in their search for suitable candidates. This is why the sum of unemployed and vacancies is taken for normalisation purposes. The dynamics of $I^{EA}$ do not change substantially if the number of unemployed is used instead.

6 They are available not via Netmind but through a special data delivery after a formal request at the FEA’s statistical service. Unfortunately, there is no access to data on the free text entered in the search masks with respect to the desired occupation or the applicant’s educational attainments and skills due to data protection restrictions.

7 This can be seen when putting the absolute effects outside the brackets.

8 The share never drops below 32% and never exceeds 34%.

9 See https://data.oecd.org/benwage/benefits-in-unemployment-share-of-previous-income.htm.

10 However, linking the VerBIS data to other data sets to control for the composition of the placement agents using VerBIS is not (yet) possible.
